# Anomalies and Curiosities of Medicine

**Published:** 1898-03

**Authors:** 


### Reviews of Books.

**Anomalies and Curiosities of Medicine.** By GEORGE M. GOULD, M.D., and WALTER L. PYLE, M.D. Pp. 968. London: The Rebman Publishing Co. (Ltd.). Philadelphia: W. B. Saunders. 1897.

This unique volume is the product of immense research. No such storehouse of extraordinary histories has ever been formed in recent times. Yet it is from the study of such residual phenomena, which seem to be exceptions to the ordinary laws of science that much of the advance of knowledge is always due. They supply valuable indications of the deficiencies in existing generalisations, and of the wide field of science which lies beyond. As Bacon says, "histories of the works of Nature which have a digression and deflexion from the ordinary course" are one of the means for the progress of science; but, as we know, he complained that in his day "a substantial and severe collection of the heteroclites or irregulars of Nature, well examined and described, I find not." We have here a praiseworthy effort to form such a collection for medical science. Almost any section might be expanded into a separate treatise, and we may add that almost every section is a good *résumé* of what is known on the subject. Still, it is beyond the power of any individual to give an exhaustive account of all known facts on so many subjects, even though he were an Aristotle himself, and a critic must not be thought ungrateful who points out missing records and tries to add his mite to the store.

Amid so much that is valuable, we must protest against space being wasted in purveying "for the appetite of vain and curious wits, as the manner of Mirabilaries is to do." The American habit of dilating upon sexual abnormalities, however unimportant, detracts from the scientific value of the book, and this blemish is the more distasteful in a beautifully illustrated volume like the present. Apart from these unfortunate paragraphs, the book is a mine of interest from beginning to end.

There are very few Americanisms and misprints. We do not know whether to "duplicate" a feat is to repeat it (page 424). "Camptown" is not the name by which the seat of the late plague epidemic—"Canton, in China"—is known. Poor Laura Bridgman, the blind deaf and dumb girl is described on page 434 as having shown an "affiliation" for her special friends, by which we presume an affection is only meant. Still these are very slight slips in such a large work.

The sections where the account of Laura is given are some of the best in the book. Other instances of persons similarly affected, of scientific interest to the psychologist, are recorded, as well as prodigies of memory, where we think some of Lord Macaulay's own feats are worth noticing. The records of the endurance of high temperature have physiological importance, but the capacity of the human race for enduring cold with little or no clothing is not less remarkable. The Roman poet's account of certain naked British tribes spending much of their time in the icy water of their streams, and Darwin's description of the Fuegians and their infants naked under the snow-storms confirm the scanty accounts here given. A fascinating history of the children brought up by wolves in India supplies the authorities on which some of Rudyard Kipling's wildest stories are founded, and a surprising number of instances are recorded.

We have compared the collection of athletic records with English ones, and found them carefully put together, allowing for the histories of one or two performances not altogether accepted on this side the water. The battle as to the existence of centenarians has been completely fought out; but it has been left for the authors to collect a vast number of instances, many of them indeed apocryphal, though others, like that of Dr. Salmon, beyond all doubt. In the stories of suspended animation, it is curious to see our old friend—the account of a lady buried with her rings, and revived by thieves pulling them off by their teeth—referred to definite cases in France and Germany; but it appears in the folklore of many countries, and has a home in one or more English churchyards. For the honour of the "ould" country too, the late Irish M.P. who was without legs and arms and yet rode to hounds, should be noticed among the armless painters and athletes.

It is difficult to select the best from such a mass of records; but the accounts of injuries of the skull seem remarkably complete and well put together, and, indeed, the descriptions of recent surgical operations are beyond all praise. We miss, however, among ovariotomies on the aged, Mr. Bush's Bristol case. There is a curious little misprint on page 659, where a British ship, the *Tartar*, is called a Tartar vessel, which reminds us of the seacoast of Bohemia; and, again, the Queen's youngest son is spoken of as "the old Duke of Albany" on page 815.

It is a great pleasure to get full histories of well-known persons like Alexis St. Martin, the man with an opening into his stomach, on whom so many theories of digestion have been based, and most readers will be surprised to hear that he was still living in 1879; but since then both he and Laura Bridgman have passed away, though Binet's case of double personality, Felida X., is still among us. The psychological notes might have been enriched with many instances given by Sir William Hamilton, among which we can only refer to the man of double personality, who for years had a continuous and connected dream by night, during which he seemed to be a rich and prosperous citizen, while in the day he toiled as a wretchedly poor labourer. The revival too, in a fever, of long chapters of Hebrew heard casually years before by a German servant is another of Hamilton's historical illustrations well worthy of record. But when all is said, we cannot but be grateful for the stupendous labour which has produced such an excellent collection, the interest and utility of which can hardly be exaggerated.

